# UP256 Inhibits Hyperpigmentation by Tyrosinase Expression/Dendrite Formation via Rho-Dependent Signaling and by Primary Cilium Formation in Melanocytes

**DOI:** 10.3390/ijms21155341

**Published:** 2020-07-28

**Authors:** Min Cheol Kang, Jae-Wook Lee, Taek Hwan Lee, Lalita Subedi, Hussain M. Wahedi, Seon-Gil Do, Eunju Shin, Eun-Yi Moon, Sun Yeou Kim

**Affiliations:** 1 College of Pharmacy, Gachon University 191, Hambakmoero, Yeonsu-gu, Incheon 21936, Korea; mincjf07@gmail.com (M.C.K.); subedilali@gmail.com (L.S.); 2 Department of Bioscience and Biotechnology, Sejong University, Seoul 05006, Korea; wodnrgo@naver.com; 3 College of Pharmacy, Yonsei University, Songdo-dong, Yeonsu-gu, Incheon 21936, Korea; lth0717@naver.com; 4 Department of Biological Sciences, National University of Medical Sciences, Mall Road, Rawalpindi 46000, Pakistan; hmwahedi@gmail.com; 5 Wellness R&D Center, Univera, Inc., Seoul 04782, Korea; sgildo@univera.com (S.-G.D.); ejayshin@univera.com (E.S.); 6 Gachon Institute of Pharmaceutical Science, Gachon University, Yeonsu-gu, Incheon 21936, Korea

**Keywords:** UP256, hyperpigmentation, tyrosinase, dendrite, ciliogenesis, melanogenesis

## Abstract

Skin hyperpigmentation is generally characterized by increased synthesis and deposition of melanin in the skin. UP256, containing bakuchiol, is a well-known medication for acne vulgaris. Acne sometimes leaves dark spots on the skin, and we hypothesized that UP256 may be effective against hyperpigmentation-associated diseases. UP256 was treated for anti-melanogenesis and melanocyte dendrite formation in cultured normal human epidermal melanocytes as well as in the reconstituted skin and zebrafish models. Western blot analysis and glutathione S-transferase (GST)-pull down assays were used to evaluate the expression and interaction of enzymes related in melanin synthesis and transportation. The cellular tyrosinase activity and melanin content assay revealed that UP256 decreased melanin synthesis by regulating the expression of proteins related on melanogenesis including tyrosinase, TRP-1 and -2, and SOX9. UP256 also decreased dendrite formation in melanocytes via regulating the Rac/Cdc42/α-PAK signaling proteins, without cytotoxic effects. UP256 also inhibited ciliogenesis-dependent melanogenesis in normal human epidermal melanocytes. Furthermore, UP256 suppressed melanin contents in the zebrafish and the 3D human skin tissue model. All things taken together, UP256 inhibits melanin synthesis, dendrite formation, and primary cilium formation leading to the inhibition of melanogenesis.

## 1. Introduction

Hyperpigmentation is a skin pigmentation disorder that is discolored, blotchy, or darker than normal skin. Hyperpigmentation occurs by an excess production of melanin attributed to multiple causes such as age, inflammation, hormone imbalance, and environmental exposure and ultraviolet (UV) [1]. Skin inflammation, burns, wounds, cuts, and any other skin injury, can result in either hyperpigmentation or hypopigmentation [2]. These disorders are difficult to treat and lack the appropriate therapeutic regimens.

Melanin is synthesized by the melanocyte, within melanosomes that are subcellular lysosome-like organelles. Melanin is important for valuable functions like determining skin appearance, and enhancing the body’s defense against the harmful effects of UV radiation [3]. Increased melanin pigmentation generally develops several major steps in the epidermis: proliferation of melanocyte, synthesis and activation of tyrosine, and transfer of the pigments from melanocyte to keratinocyte [4]. Melanocyte is a specific dendritic cell that produces melanin via a process called melanogenesis [5]. Melanin synthesis is regulated by numerous enzymes such as tyrosinase and tyrosinase-related protein 1 and 2 (TRP-1, TRP-2). Activation of tyrosinase converts tyrosine to melanin [6], and melanogenic enzyme transcription is controlled by the microphthalmia-associated transcription factor (MITF) [7,8]. SOX9 also regulates MITF, dopachrome tautomerase, and tyrosinase promoters, leading to an increase in the expression of these key melanogenic proteins, and finally to a stimulation of pigmentation [9].

Melanin skin pigmentation is tightly linked to the transfer of melanin-containing melanosomes from melanocytes to neighboring keratinocytes [10]. Melanocytes have many dendrites, allowing for continuous contact with the nearby keratinocytes. The long and extended melanocyte dendrites are essential for melanosome trafficking during pigment transfer [11]. These endosomal organelles are moved from the central cell body to the dendritic tips, their final destination for transportation. The members of the Rho family of small GTP-binding proteins act as master regulators of dendrite formation [12]. The small GTP-binding proteins CDC42, Rac, and Rho regulate melanocyte dendricity in response to hormones and ultraviolet irradiation [13].

The determination of safe and effective candidate compounds that can attenuate hyperpigmentation is of great necessity. Cosmeceuticals are commonly used for reducing melanin content; these agents target hyperplastic melanocytes selectively, and prevent major regulatory steps in the synthesis of melanin [14]. A recent study has suggested that oxidative stress reduction, inflammatory mediator production, rate of melanin synthesis, and hormonal balance might control melanin production [15]. Many studies have identified the agents involved in regulating melanin synthesis. A number of botanical and natural ingredients have been reported as depigmenting products. Arbutin, kojic acid, and azelaic acid inhibit melanogenesis by controlling tyrosinase activity. Sphingosin-1-phosphate and ceramides could induce MITF degradation or block MITF expression, leading to a reduction of tyrosinase production. Several topical fatty acids including linoleic acid showed a depigmenting effect on guinea pig skin after UV exposure. Niacinamide was demonstrated to inhibit melanosome transfer to keratinocytes [16,17,18]. However, some of them have presented with various side effects such as cytotoxicity, dermatitis, and erythema, specifically after long-term use. Therefore, it is important to find a treatment regimen capable of skin depigmentation, but void of adverse effects either directly (the skin) or to other body parts.

Bakuchiol (1-(4-hydroxyphenyl)-3,7-dimethyl-3-vinyl-1,6-octa-diene), a monoterpene phenol from Psoralea corylifoia seeds, is a well-known medication against acne vulgaris. According to previous studies, bakuchiol has anti-oxidant, anti-microbial, and anti-cancer activities [19,20,21]. UP256 is an active ingredient containing bakuchiol, and is used for treating acne vulgaris [22]. Post-inflammatory hyperpigmentation such as in acne, can cause skin darkening and discoloration that shows up as spots or large unpleasant patches. We hypothesized that UP256 may be effective in treating hyperpigmentation, since dark spots on the skin are also caused by acne. It would be great compliance if a medicine or cosmeceutical agent could double as a treatment for inflammatory as well as undesirable hyperpigmentation conditions. In this study, we investigated the anti-melanogenic effect of UP256 containing bakuchiol, and its mechanism of action on melanogenesis in melanocytes. We also confirmed the anti-melanogenic effect of UP256 in 3D skin tissue and zebrafish embryos.

## 2. Results

### 2.1. Inhibitory Effects of UP256 on Melanin Synthesis in Melanocytes

**Figure 1:**
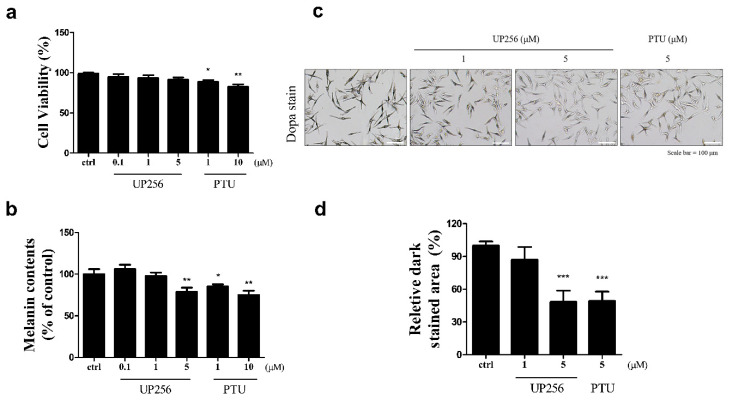
Effects of UP256 on melanogenesis in NHEM. Cell viability (**a**) and melanin content were measured (**b**) after treatment with UP256 (0.1, 1, and 5 µM) for 72 h. PTU was used as a positive control. In situ tyrosinase activity in NHEMs was observed via L-DOPA staining (**c**). Scale bar = 100 µm. Relative amounts of stained area were measured with the ImageJ program. The results are calculated as a percentage of the vehicle-treated control (**d**) and expressed as mean ± SD of three independent experiments (* *p* < 0.05 ** *p* < 0.01, and *** *p* < 0.001, compared with the vehicle-treated control).

We investigated the effect of UP256 on melanin synthesis in normal human epidermal melanocytes (NHEM). Well-known melanogenesis inhibitor, phenylthiourea (PTU) [23], a positive control, was cytotoxic at both treatment concentrations (1 and 10 µM), while UP256 had no significant cytotoxicity at its effective concentrations up to 5 µM (Figure 1a). UP256 (5 µM) reduced melanin production in NHEMs by 15%. At 5 µM, the potency of UP256 appeared almost similar to PTU at 10 µM (Figure 1b). We further confirmed the anti-melanogenic effect of UP256 using L-3,4-dihydroxyphenylalanine (L-DOPA) staining, which detects in situ tyrosinase activity (Figure 1c). UP256 treatment at 5 µM significantly inhibited melanin synthesis, similar to PTU at the same concentration (Figure 1d). UP256 showed an inhibitory effect on cellular tyrosinase activity (Figure S1). In contrast, under cell-free conditions, UP256 did not show significant inhibitory effect on the activity of mushroom tyrosinase (Figure S2), suggesting that the effect on melanocytes was not mediated by direct inhibition of UP256 with the tyrosinase enzyme.

### 2.2. Effects of UP256 on the Expression of Melanogenic Enzymes in Melanocytes

**Figure 2:**
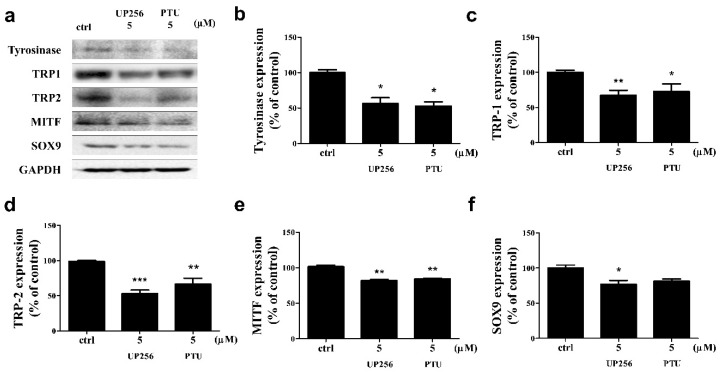
Effects of UP256 on the expression of melanogenic pathway proteins. NHEMs were treated with 5 µM of UP256 and PTU for 72 h and analyzed using western blotting (**a**). Observed protein expression of tyrosinase (**b**), TRP-1 (**c**), TRP-2 (**d**), MITF (**e**), and SOX9 (**f**). The band intensities were quantified, and the integrated areas normalized, first to the corresponding value of GAPDH, and then to the signal observed in the vehicle-treated control. All data are presented as mean SD of three independent experiments. * *p* < 0.05, ** *p* < 0.01 and ***p < 0.001 compared with the vehicle-treated control.

Tyrosinase, TRP-1, TRP-2, and SOX9 are simultaneously regulated by each other during melanogenesis. We observed the effect of UP256 on the expression of these melanogenic enzymes via western blot analysis (Figure 2a). UP256 significantly inhibited the expression of tyrosinase (approximately −43%), TRP-1 (−32%), TRP-2 (−47%), MITF (−19%), and SOX9 (−23%) at 5 µM, compared to the vehicle-treated cells after 72 h of treatment. UP256 reduced the expression of tyrosinase and MITF similarly, but it reduced TRP-1, 2, and SOX9 more effectively, compared to PTU (Figure 2b–e).

### 2.3. Effects of UP256 on Rac1, Cdc42, and α-PAK Signaling Proteins in Melanocytes

**Figure 3:**
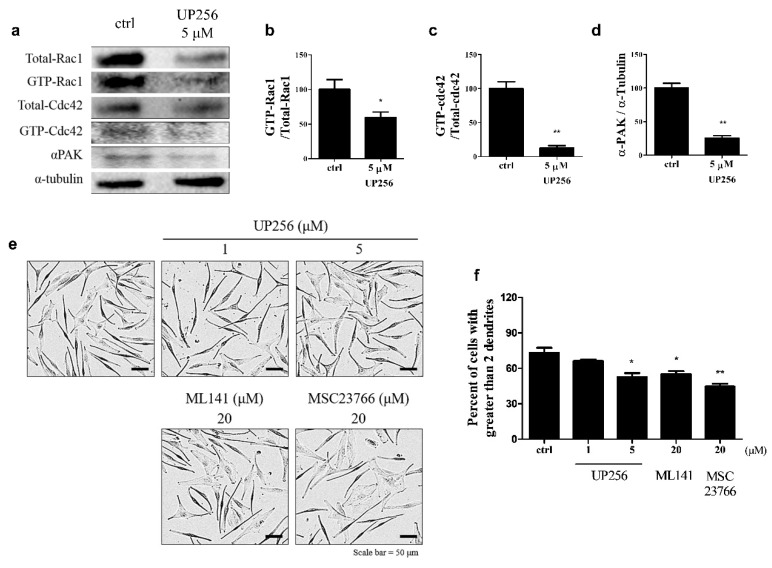
Analyses of the effects of UP256 on the expression of proteins responsible for melanocyte dendrite formation (**a**). The expression of GTP-bound Rac1 (**b**), Cdc42 (**c**), and α-PAK (**d**) was measured using western blotting. The number of cells with more than two dendrites was counted in pictures (**e**) and represented as the percentage of total cells (**f**). A total of 300 cells were counted from each experimental group. Scale bar = 50 µm. All data are presented as mean SD of three independent experiments. * *p* < 0.05 and ** *p* < 0.01 versus the vehicle-treated control.

To determine the effect of UP256 in the regulation of small GTP-binding proteins related to dendrite formation in melanocytes, we performed a pull-down assay for cellular GTP-Rac1 and Cdc42. As shown in Figure 3a–d, UP256 treatment markedly inhibited Rac1 and Cdc42 activation, and decreased α-PAK expression. These data suggest that the inhibition of the GTP-Rac1, GTP-Cdc42, and α-PAK pathways is involved in UP256-induced inhibition of dendrite formation. We used ML141, a Cdc42 inhibitor, and selective MSC 23766, a selective Rac1 inhibitor, to further confirm our findings. As expected, the inhibitor treatment (20 μM) decreased the dendrite levels in a manner similar to UP256 treatment (Figure 3e,f).

### 2.4. Inhibitory Effects of UP256 on Cilia Formation in Melanocytes

**Figure 4:**
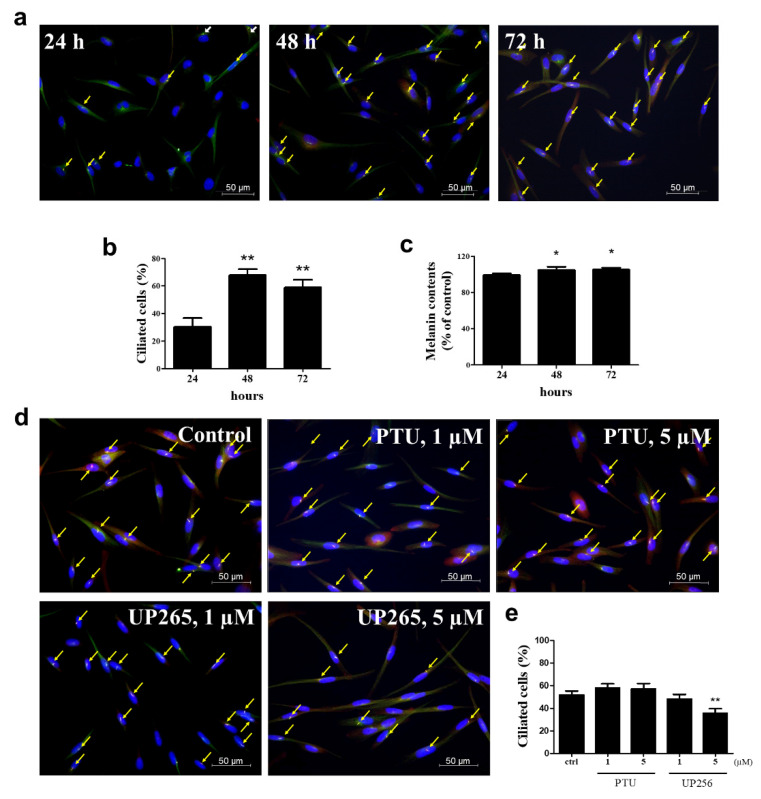
Analyses of primary cilia formation. (**a**) NHEM cells were incubated on cover glass for 24, 48, and 72 h. The cells were fixed and stained with antibody against Ac-tubulin (green) or Arl13b (red) and DAPI (blue). (**b**) The ciliated cells out of more than 500 cells were counted at the image overlaid with three fluorescence. Processing (such as changing brightness and contrast) was applied equally to the controls across the entire image. (**c**) Melanin contents. (**d**) NHEM cells were incubated on cover glass in the presence of 1 and 5 µM UP256 or PTU as a positive control for 72 h. Three fluorescence were overlaid to observe the primary cilium in each cell. Yellow arrows indicate the primary cilia in the representative fluorescence image for each group. (**e**) The ciliated cells out of more than 500 cells were counted at the image overlaid with three fluorescence. * *p* < 0.05 and ** *p* < 0.01.

To investigate the relationship with melanogenesis and cilia formation, we measured the cilia formation and melanin contents of melanocytes were increased over time (24, 48, and 72 h) (Figure 4a). As shown in Figure 4b,c, we observed that the melanin slightly increased as time passed, and the formation of cilia also increased at 48 and 72 h. We also measured cilia formation in the sample treatment group (Figure 4d). UP256 treatment decreased approximately 20% of cilia formation in melanocytes compared to the control group, but PTU treatment did not change the cilia (Figure 4e).

### 2.5. Effects of UP256 on Melanogenesis In Vivo and Ex Vivo

**Figure 5:**
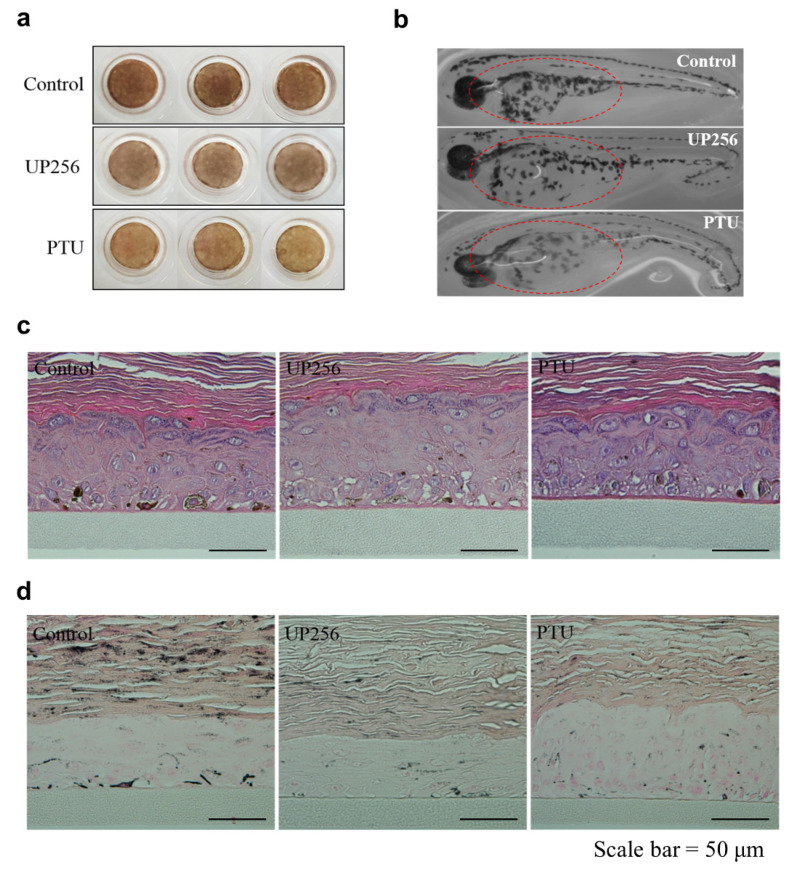
Depigmentation effects of UP256 in reconstructed skin and zebrafish. A total of 0.05% UP256 (*w*/*v*) and 0.1% PTU (*w*/*v*) were applied, respectively, to the reconstructed skin tissue for 14 days. Following the incubation period, the tissue was photographed using a digital camera (**a**). Synchronized zebrafish embryos were treated with 30 μM UP256 and PTU and observed under a stereomicroscope after 72 h (**b**). Reconstructed tissues were fixed and stained with H&E (**c**) and Fontana-Masson silver stains (**d**). Scale bar = 50 µm.

We tested the effect of UP256 on melanogenesis in the zebrafish model as well as the reconstructed skin tissue. UP256 clearly inhibited melanogenesis in the 3D skin model (Figure 5a). No UP256-induced toxicity was seen in hematoxylin and eosin (H&E) staining (Figure 5c). Moreover, melanin production was lower in the UP256-treated group, compared with the control group (Figure 5d). We also determined whether UP256 was effective in vivo by treating the zebrafish embryo model with UP256 for 72 h. We observed that UP256 inhibited melanogenesis in the melanocytes as well as in the zebrafish embryos (Figure 5b). The melanin amount in zebrafish was slightly decreased after treating the UP256 sample when compared to the control group, but not as much as the PTU group (Figure S3). In the 3D human skin model, UP256 highly inhibited the melanin level similar to the positive control. In our 3D human skin model, the analysis showed that USP256 had better depigmenting activity compared to the zebra fish model.

## 3. Discussion

In our study, we attempted to highlight the possible use of UP256, a natural product, for treating hyperpigmentation and associated diseases. Pigmentation can be regulated following several steps: regulation of melanin synthesis, melanosome transfer to other epidermal cells, and melanosome degradation and turnover [5]. Synthesis of melanin takes place in specialized intracellular organelles called melanosomes, catalyzed by melanogenesis enzymes such as tyrosinase. Mature melanin-filled melanosomes move from the perinuclear region to the dendrite tips of melanocytes. Effective regulation of melanin synthesis is crucial in skin whitening and treating hyperpigmentation.

It appears that the reduced level of melanin in NHEMs after treatment with UP256 is due to decreased melanogenic enzyme expression. Melanin synthesis is initiated by hydroxylation of tyrosine to L-DOPA and further conversion of L-DOPA to DOPA-quinone, which acts as a precursor molecule for melanin synthesis via various pathways [24]. Tyrosinase is a crucial enzyme involved in the transformation of L-tyrosine to L-DOPA [25]. Melanocytes were stained with L-DOPA, which detects in situ tyrosinase activity and is a more sensitive indicator of changes in melanin synthesis than the determination of total melanin levels. Our data showed that UP256 inhibited the melanin content in melanocytes. We further confirmed the depigmenting effect of UP256 by the reduced tyrosinase expression under the same conditions. Two other proteins, TRP-1 and TRP-2, are known as supportive enzymes for tyrosinase [26]. TRP-1 plays an important role in tyrosinase activation and stabilization. TRP-1 further helps to increase melanosome synthesis, and the eumelanin/pheomelanin ratio [27]. Similarly, TRP-2 acts as a Dct that helps eumelanin synthesis, especially via the dopachrome [24]. UP256 inhibits TRP-1 and TRP-2 protein expression, leading to a reduction in their supportive role in melanin synthesis. The reduction in the levels of melanin after treatment with UP256 appears to be a result of a decrease in the expression of melanogenic enzymes, probably due to a reduced expression of MITF. UP256 inhibits expression of transient receptor potential cation channel subfamily M member 1 (TRPM1; also known as melastatin) which is controlled by MITF. Activation of TRPM1 leads to induce the melanogenesis as well as differentiation of melanocytes and UP256 inhibited TRPM1 mRNA expression (Figure S4). Additionally, SOX9 is also associated with melanogenesis, melanocyte differentiation, and skin pigmentation [28]. SOX9 specifically controls the function of Dct, the tyrosinase promoter, leading to highly stimulated pigmentation upstream [29]. Our results showed that UP256 decreased SOX9 expression, followed by a reduction in TRP-1, TRP-2, and tyrosinase, resulting in low melanin production in NHEMs.

In melanocytes, melanosomes mature and are trafficked to dendritic tips, where they are transferred to adjacent epidermal keratinocytes through pathways that involve microtubule networks and the actin cytoskeleton. Melanocytic dendrite formation and extension are the foremost steps for melanosome transfer to nearby keratinocytes. Therefore, dendrite formation is critically important for melanosome transfer. GTP-binding proteins regulate cytoskeletal organization including dendritic formation. In particular, Rho family GTPases including Cdc42, Rac1, and RhoA play a vital role in the process of melanocyte dendrite formation and extension. Rac1 has been known to activate dendrite and lamellipodia formation, while Cdc42 is involved in filopodia and outer neurite formation [30]. Given the role of Cdc42 and Rac1 in cytoskeletal organization, it is convincing that UP256 decreases Cdc42 and Rac1 activation and induces the reduction of dendrite formation. Additionally, Rac1-α-PAK signaling is a well-known link for the dendritic spine formation, and their crosstalk also helps in melanocyte dendrite formation [31]. In our study, UP256 downregulated dendrite formation, because it inhibited GTP-Rac1, GTP-Cdc42, and α-PAK, and ultimately inhibited melanosome transfer. Our data confirm that UP256-mediated dendrite formation inhibition is similar in effect to that of the well-known Rho family protein inhibitors: ML141 (Cdc42 inhibitor) and NSC 23,766 (Rac1 inhibitor). Therefore, it is clear that UP256 regulates Rho-GTP-binding proteins, especially Rac1 and Cdc42, all critical for dendrite formation. The upstream signaling intermediates that regulate Rac1 and RhoA activity require more extensive studies as well as the potential crosstalk between dendrites and the keratinocyte membrane at the attachment site.

It is contradictory regarding the relationship between melanogenesis and cilia formation when we measured the cilia formation and melanin contents in NHEM over time (24, 48, and 72 h) (Figure 4a), Interestingly, we found that the formation of cilia increased at 48 and 72 h passed with time, but the melanin was slightly increased at that time. It is reported that cilia might bear out by several shocks and stress, and UV and heat shock both triggered cilia assembly in RPE-1 cells [32,33]. Melanocytes are more sensitive to UVR-damage than any other cells. Therefore, melanocytes may play an important role in cell signal processing and melanin synthesis through cilia. However, melanin synthesis by exposure to α-MSH was significantly reduced by the induction of primary cilium formation with cytochalasin D (CytoD), and melanin was significantly elevated by treatment with ciliobrevin A (Cilio A), an inhibitor of primary cilium formation [34]. This indicated that melanogenesis could be inhibited by the ciliogenesis enhanced by cytoskeleton depolymerization with CytoD. However, little has been informed about how the ciliogenesis could be increased in inactive cells without the cytoskeleton dynamics with CytoD. Our data demonstrated that melanogenesis could be correlated with ciliogenesis based on the incubation time of NHEM and suggests that the treatment of CytoD might inhibit melanogenesis just by regulating the actin polymerizations independently in primary cilia formation. Therefore, there may be limitations to generalize the relationship with melanogenesis and cilia formation. In contrast, our results showed that treatment of UP256 significantly decreased cilia formation. Our results were tested under normal conditions where function was maintained the same in in vivo. Therefore, our results suggest that the depigmenting effect by UP256 may be due to decreasing the dendrite movement via regulating the cilium cytoskeleton dynamics in melanocytes.

The zebrafish is an excellent and well-established model for in vivo studies involving pigmentation experiments. Based on the inhibitory effect of UP256 on melanogenesis in NHEMs, zebrafish model studies were performed to confirm the effect of UP256 on hyperpigmentation. An additional study was conducted to confirm the effectiveness of the newly discovered anti-melanogenesis inhibitors in an artificial skin model. Histological changes resulting from UP256-induced depigmenting were observed using Fontana-Masson staining.

UP256 has a potential inhibition effect on melanin production by reducing tyrosinase, TRP-1 and -2, and SOX-9 expression in melanocytes. This anti-melanogenic effect was further confirmed in a 3D skin model and zebrafish. In addition, UP256 has an apparent regulatory role in melanocyte dendrite formation. Therefore, we conclude that UP256 is a promising potential therapeutic agent for hyperpigmentation-related skin diseases and skin freckles.

## 4. Materials and Methods

### 4.1. Preparation of UP256

UP256 was provided by Univera, Inc (Seoul, Korea). UP256 is a natural bakuchiol extract composition comprised of 77.02% bakuchiol, and <100 ppm furanocoumarin prepared from *Psoralea corylifolia* L. seeds using the patented process (US 20160022602 A1).

### 4.2. Cell Culture and Treatments

Normal human epidermal melanocytes (NHEM) cells (Lonza, Walkersville, MD, USA) were cultured in Melanocyte Medium plus Bullet Kit (Lonza, Walkersville, MD, USA) at a humidified atmosphere of 5% CO_2_ at 37 °C. Cells were treated in the presence or absence of UP256 or PTU for 24 h. Additionally, where necessary, cells were pre-incubated with either the Cdc42 inhibitor (ML141, Sigma-Aldrich, St Louis, MO, USA) or the Rac1 inhibitor (MSC 23766, Tocris Bioscience, UK).

### 4.3. Cell Viability and In Situ Tyrosinase Activity

Cell viability was performed as previously described [29]. Briefly, cells were incubated with 0.1% (*w*/*v*) 3-(4, 5-dimethylthiazol-2-yl)-2,5-diphenyltetrazolium bromide reagent for 1 h. The resulting formazan crystals were then dissolved in dimethyl sulfoxide (DMSO), and the absorption measured at 570 nm, using a microplate reader from Molecular Devices (San Jose, CA, USA). To evaluate in situ tyrosinase activity, L-DOPA staining was performed, following the method reported [30]. Briefly, NHEM were fixed with 4% paraformaldehyde and permeabilized using the 0.1% Triton X-100 reagent. Cells were stained with 0.1% L-DOPA for 3 h at 37 °C, and observed under a microscope (Olympus, Tokyo, Japan).

### 4.4. Western Blot Analysis

NHEM were treated with UP256 or PTU for 24 h. The cells were then rinsed with phosphate-buffered saline (PBS), lysed with Pro-PrepTM solution (iNtRON Biotechnology; Seoul, Korea), and lysates were centrifuged at 12,000 rpm for 30 min. Total protein in the supernatant was estimated using a Bio-Rad Bradford Assay Kit (Hercules, CA, USA). The proteins were separated using sodium dodecyl sulfate-polyacrylamide gel electrophoresis (SDS-PAGE) and transferred onto a polyvinylidene difluoride (PVDF) membrane. The membrane was blocked with 5% skim milk in tris-buffered saline (TBS) containing 0.05% Tween-20 (TBST) buffer, and then incubated with Tyrosinase, TRP-1, TPR-2, and SOX9 antibodies (Abcam, Cambridge, MA, USA), and Rac1, Cdc42, and α-PAK antibodies (Santa Cruz Biotechnology, Santa Cruz, CA, USA) for overnight at 4 °C. Immunoreactive bands were visualized with the Pierce ECL western blotting substrate (Thermo Scientific; Rockford, IL, USA), using ChemiDoc (BioRad Laboratories, Hercules, CA. USA).

### 4.5. Zebrafish Model

Zebrafish embryos obtained from the Zebrafish Resource Bank (Kyungpook National University, Daegu, South Korea) were treated with UP256, with PTU as a positive control, for 9–72 h post-fertilization. Treated zebrafish embryos appeared more depigmented compared to the vehicle-treated embryos. This depigmentation was clearly revealed when we observed the zebrafish under a stereomicroscope.

### 4.6. 3D Tissue Model

MelanoDerm^TM^ is a reconstructed skin that was purchased from MatTek (Ashland, MA, USA), and consists of human keratinocytes and human melanocytes cultured to form a multilayered, well-differentiated model of the human epidermis. Reconstructed skin tissue was cultured with EPI-100-NMM medium (MatTek, Ashland, MA, USA) under conditions of 5% CO_2_ at 37 °C. UP256 was dissolved in a mixture of propylene glycol and PBS (50:50, *v/v*), and then treated to skin tissue every two days. The tissues were rinsed with PBS, fixed with 10% Neutral Buffered Formalin, and embedded in paraffin and sectioned at 4 µm. The sections were stained with Hematoxylin–Eosin (H&E) and Fontana-Masson and then the stained slides were examined under a light microscope (Olympus, Tokyo, Japan).

### 4.7. Detection of Primary Cilia

For the detection of primary cilia in vitro (Lee et al., 2019), NHEMs were grown on a coverslip and then incubated for 24–72 h. Cells were fixed with 4% paraformaldehyde for 10 min, washed three times with cold PBS, and permeabilized with PBST (0.1% (*v/v*) Triton X-100 in PBS) for 10 min. Then, cells were washed three times, and incubated with monoclonal anti-acetylated tubulin antibodies (1:1000, Sigma-Aldrich St. Louis, MO, USA) and rabbit Arl13b antibodies diluted (1:1000, Rosemont, IL, USA) in PBST for 1 h at room temperature. After washing three times with PBS, cells were incubated with chicken anti-mouse IgG-Alexa 488 diluted (1:1000, Life technology, Carlsbad, CA, USA) and anti-Rabbit-Alexa 568 (1:2000, Life technology, Carlsbad, CA, USA) in PBST for 1 h at room temperature. Nucleus was visualized by staining cells with DAPI. After washing with PBS, cells were mounted on a glass slide. Primary cilia were observed and photographed at 400x magnification under a fluorescence microscope (Nikon, Tokyo, Japan).

### 4.8. Statistical Analyses

The results were analyzed using Bonferroni’s test for multiple comparisons of one-way analysis of variance (ANOVA) using GraphPad Prism 5.0 (GraphPad Software Inc., San Diego, CA, USA). *p*-values of <0.05, <0.01, and <0.001 were considered statistically significant. Results are presented as the mean and the standard error of the mean (SEM).

## 5. Conclusions

For the first ime, we discovered that UP256 containing bakuchiol could inhibit melanin synthesis, dendrite formation, and primary cilium formation leading to the inhibition of melanogenesis and the mechanism might involve the regulation of the expression of Tyrosinase Expression and Rho-Dependent Signaling. Therefore, we conclude that UP256 is a promising potential therapeutic agent for hyperpigmentation-related skin diseases.

## Supplementary Materials

The following are available online at https://www.mdpi.com/1422-0067/21/15/5341/s1, Figure S1. Effects of UP256 on the cellular tyrosinase activity in melanocytes. Figure S2. Effects of UP256 on mushroom Tyrosinase activity. Figure S3. Relative melanin content in the zebrafish embryos. Figure S4. Effects of UP256 on expression of Trpm1 mRNA in melanocytes.
