# Supplementary material for: UP256 Inhibits Hyperpigmentation by Tyrosinase Expression/Dendrite Formation via Rho-Dependent Signaling and by Primary Cilium Formation in Melanocytes
Source: Int J Mol Sci. 2020 Jul 28;21(15):5341. doi: 10.3390/ijms21155341 (PMC7432859; doi:10.3390/ijms21155341)
Supplement: Supplementary file 1 [file ijms-21-05341-s001.pdf]

International Journal of
Molecular Sciences

MDPI

Supplementary:

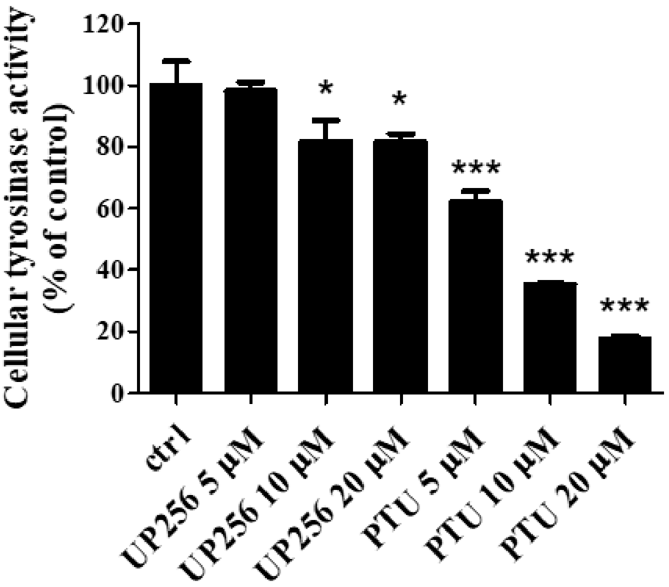

Figure S1. Effects of UP256 on the cellular tyrosinase activity in melanocytes. after incubation, tyrosinase activity was evaluated using l-DOPA oxidation assay

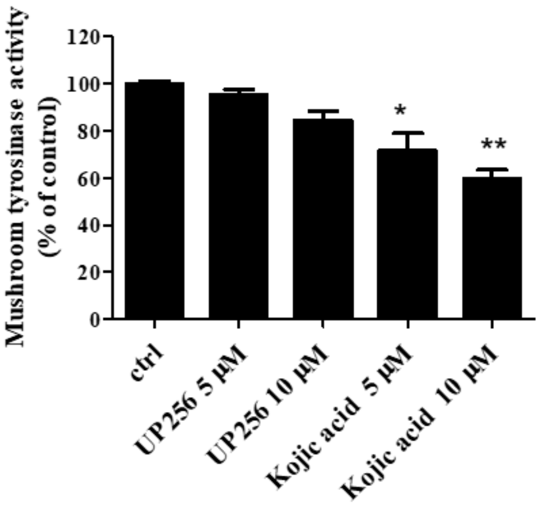

Figure S2. Effects of UP256 on mushroom Tyrosinase activity. For the measurement of mushroom Tyrosinase activity, sample and L-DOPA were mixed with mushroom Tyrosinase. Kojic acid was used as a positive control.

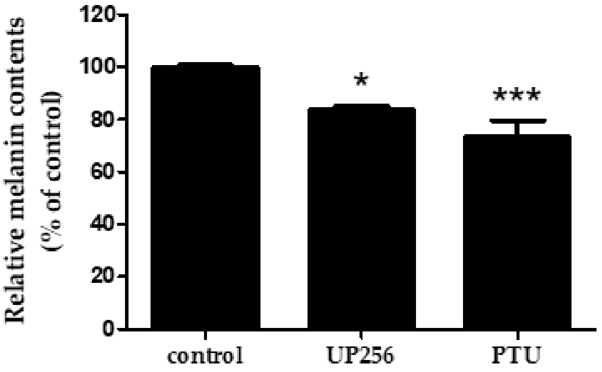

Figure S3. Relative melanin content in the zebrafish embryos. Synchronized zebrafish embryos were treated with 30 μM UP256 and PTU and observed under a stereomicroscope after 72 h.

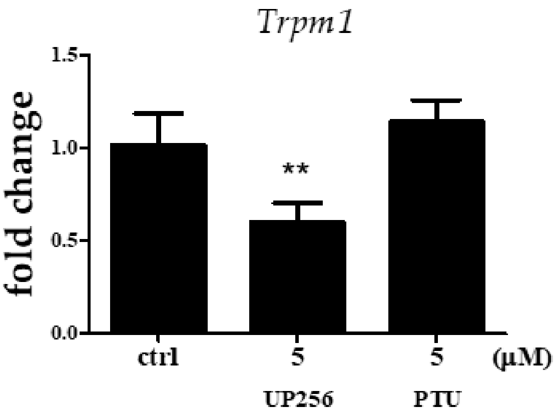

Figure S4. Effects of UP256 on expression of Trpm1 mRNA in melanocytes. Expression levels were calculated by quantitative real-time PCR analysis. GAPDH gene was used as internal reference gene and the ΔΔ CT method was used for relative quantification.
